# De novo sequencing of the transcriptome reveals regulators of the floral transition in *Fargesia macclureana* (Poaceae)

**DOI:** 10.1186/s12864-019-6418-2

**Published:** 2019-12-30

**Authors:** Ying Li, Chunxia Zhang, Kebin Yang, Jingjing Shi, Yulong Ding, Zhimin Gao

**Affiliations:** 1 State Forestry and Grassland Administration Key Open Laboratory on the Science and Technology of Bamboo and Rattan, Institute of Gene Science and Industrialization for Bamboo and Rattan Resources, International Centre for Bamboo and Rattan, Beijing, 100102 China; 2 Bamboo Research Institute, Nanjing Forestry University, Nanjing, 210037 Jiangsu China

**Keywords:** Transcriptome, Floral transition, Bamboo, Qinghai–Tibet plateau

## Abstract

**Background:**

*Fargesia macclureana* (Poaceae) is a woody bamboo species found on the Qinghai–Tibet Plateau (QTP) approximately 2000 ~ 3800 m above sea level. It rarely blossoms in the QTP, but it flowered 20 days after growing in our lab, which is in a low-altitude area outside the QTP. To date, little is known regarding the molecular mechanism of bamboo flowering, and no studies of flowering have been conducted on wild bamboo plants growing in extreme environments. Here, we report the first de novo transcriptome sequence for *F. macclureana* to investigate the putative mechanisms underlying the flowering time control used by *F. macclureana* to adapt to its environment.

**Results:**

Illumina deep sequencing of the *F. macclureana* transcriptome generated 140.94 Gb of data, assembled into 99,056 unigenes. A comprehensive analysis of the broadly, specifically and differentially expressed unigenes (BEUs, SEUs and DEUs) indicated that they were mostly involved in metabolism and signal transduction, as well as DNA repair and plant-pathogen interactions, which may be of adaptive importance. In addition, comparison analysis between non-flowering and flowering tissues revealed that expressions of *FmFT* and *FmHd3a*, two putative *F. macclureana* orthologs, were differently regulated in NF- vs F- leaves, and carbohydrate metabolism and signal transduction were two major KEGG pathways that DEUs were enriched in. Finally, we detected 9296 simple sequence repeats (SSRs) that may be useful for further molecular marker-assisted breeding.

**Conclusions:**

*F. macclureana* may have evolved specific reproductive strategies for flowering-related pathways in response to photoperiodic cues to ensure long vegetation growing period. Our findings will provide new insights to future investigations into the mechanisms of flowering time control and adaptive evolution in plants growing at high altitudes.

## Background

The flowering time is of crucial importance to ensure the reproductive success of flowering plants. Previous results have indicated that the floral transition is orchestrated by several parallel and interactive genetic pathways that are regulated by a variety of environmental and endogenous signals [1]. Many key genes and regulatory networks have been identified in herbaceous annual plants such as Arabidopsis [2, 3], rice [4], gourds [5], potato [6] and sorghum [7]. However, much less is known about such regulation in perennial plants. Despite the increasing attention on perennial dicotyledonous woody plants such as poplar [8, 9], eucalyptus [10] and citrus [11] species, to date, the molecular mechanism underlying floral regulation in monocotyledonous woody plants remains elusive. Furthermore, previous studies investigated flowering mainly by artificially altering the external signals (e.g. photoperiod and light intensity) and did not assess the impact of the original environment on the adaptive evolution of species-specific reproductive strategies.

Bamboo plants are an important group in the Bambusoideae subfamily of the monocotyledonous Poaceae. They exhibit a wide degree of variation in the timing (1–120 years) and nature (sporadic vs. gregarious) of flowering among species [12]. Sporadic flowering involves flowering in only a few isolated clumps, which set little or no seed and usually remain alive afterward [13]. In contrast, gregarious flowering involves all individuals of a species regardless of age and/or location within and among the populations at the same time, which is usually followed by death and seed setting [14]. And the simultaneous death of many individuals triggers serious ecological consequences, including changes in the population dynamics of neighboring plants, differences in soil properties, various effects on endangered animals that depend on bamboo [15], and the knock-on effects on human economies in many parts of the world [16]. Therefore, dissecting the regulators that control the unique life history of bamboo may be of use for plant ecology and human society. However, to date, little is known regarding the molecular mechanisms of bamboo flowering, in part because of the sporadic occurrence of these flowering episodes and the long intervals between events.

Many genes have been identified as regulators of reproductive development in different bamboo species, including the MADS-box transcription factors [17–19], *CONSTANS* (*CO*) [20] and *FLOWERING LOCUS T* (*FT*) [21], among others. In addition, studies of sequenced transcriptomes have identified microRNAs related to floral development [22–24]. However, samples collected in these analyses were limited to mature spikelets or to different spikelets at different development stages. Thus, it is likely that dynamic changes in genes occurring at different development stages may be missing. In addition, the specific response of particular tissues to internal and external cues and how plants integrate these signals to regulate different phases of reproductive development (including the floral transition, florigen transport, and floral organ specification) has not yet been elucidated in bamboo. Furthermore, no studies of flowering have been conducted on wild bamboo plants growing in extreme environments.

**Fig. 1:**
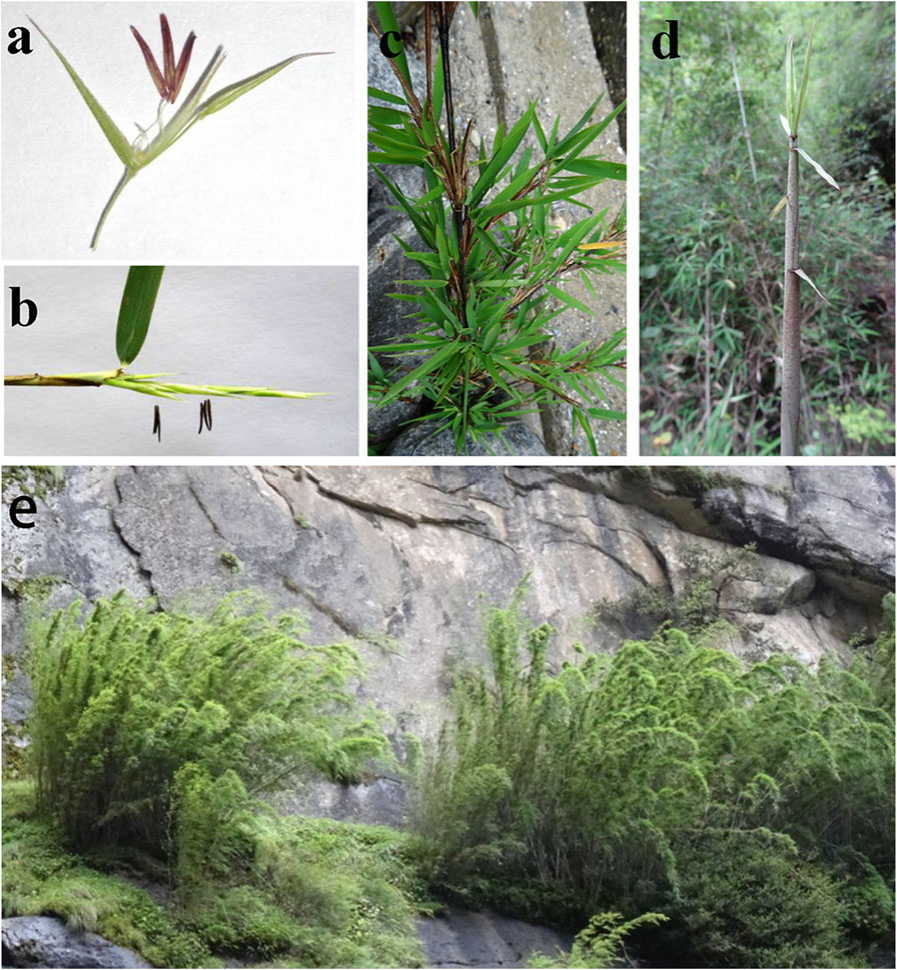
Seedlings of *Fargesia macclureana* flowered shortly after being transferred from the Qinghai–Tibet Plateau (QTP) approximately 2000 ~ 3800 m above sea level to a low altitude lab. **a**-**b** Floret and spikelet of a flowering seedling maintained at the low altitude lab outside the QTP. **c**-**d** The seedling and shoot of plants growing on the QTP. **e** The original growing environment of *F. macclureana*

Here, we took advantage of an unexpected flowering event in highland arrow bamboo, *Fargesia macclureana* [25], and performed the first de novo transcriptome analysis. This transcriptome includes data from six different tissues collected at different development stages, including inflorescences in the initial and peak flower stage (I- and P- spikelets), branchlets, and leaves from both flowering and non-flowering bamboo plants (F/NF-branchlets and F/NF -leaves). *F. macclureana* is a woody bamboo species found in areas 2000 ~ 3800 m above sea level on the Qinghai–Tibet Plateau (QTP) (Fig. 1), which is the highest and largest plateau in the world. The growth environment of the QTP is characterized by low temperature and low oxygen availability, reduced pathogen incidence, and intense radiation [26]. *F. macclureana* rarely blossoms in the QTP, but it flowered 20 days after growing in our lab, which is in a low-altitude area outside the QTP. Our goal is to use the transcriptomic data to gain a deeper understanding of the mechanisms underlying the control of flowering time and the adaptation of *F. macclureana* to the complex extreme conditions of the QTP. On one hand, we expect to detect regulatory hubs involved in the flowering mechanisms. On the other hand, we aim to discover signs of the adaptive evolutionary changes in *F. macclureana* in response to the harsh environmental conditions in the QTP, which may, in turn, provide a broader insight into the adaptive mechanisms for plants that grow at high altitudes.

## Results

### De novo transcriptome assembly yielded 99,056 unigenes

Illumina deep sequencing of the *F. macclureana* transcriptome generated 140.94 Gb of data, including 471,537,304 clean reads in 18 unique samples (Additional file 1: Table S1). The average Q20 (sequencing error rate less than 1%) and Q30 (sequencing error rate less than 0.1%) percentages were 95.64 and 89.95% respectively. The GC content of all samples ranged from 53.78 to 55.86%, with an average of 54.81%. Sample data were assembled into 289,122 transcript scaffolds, with an N50 and average length of 1765 bp and 1183 bp, respectively. The final de novo assembly included 99,056 unigenes, with an N50 and average length of 1587 bp and 926 bp, respectively. Among these unigenes, 71.02% (70,354) were shorter than 1000 bp and 12.06% (11,950) were longer than 2000 bp (Table 1).

**Table 1:** Length range of transcripts and unigenes identified in the transcriptome of *F. macclureana*

Length Range	Transcripts	Unigenes
200–300	36,390 (12.59%)	25,291 (25.53%)
300–500	47,515 (16.43%)	21,257 (21.46%)
500–1000	78,453 (27.13%)	23,806 (24.03%)
1000-2000	77,456 (26.79%)	16,752 (16.91%)
2000+	49,308 (17.05%)	11,950 (12.06%)
Total number	289,122	99,056
Total length	341,956,623	91,685,618
N50 length	1765	1587
Mean length	1182.74	925.59
Total number	289,122	99,056
Total length	341,956,623	91,685,618
N50 length	1765	1587
Mean length	1182.74	925.59

### Most unigenes were functionally annotated and classified

**Fig. 2:**
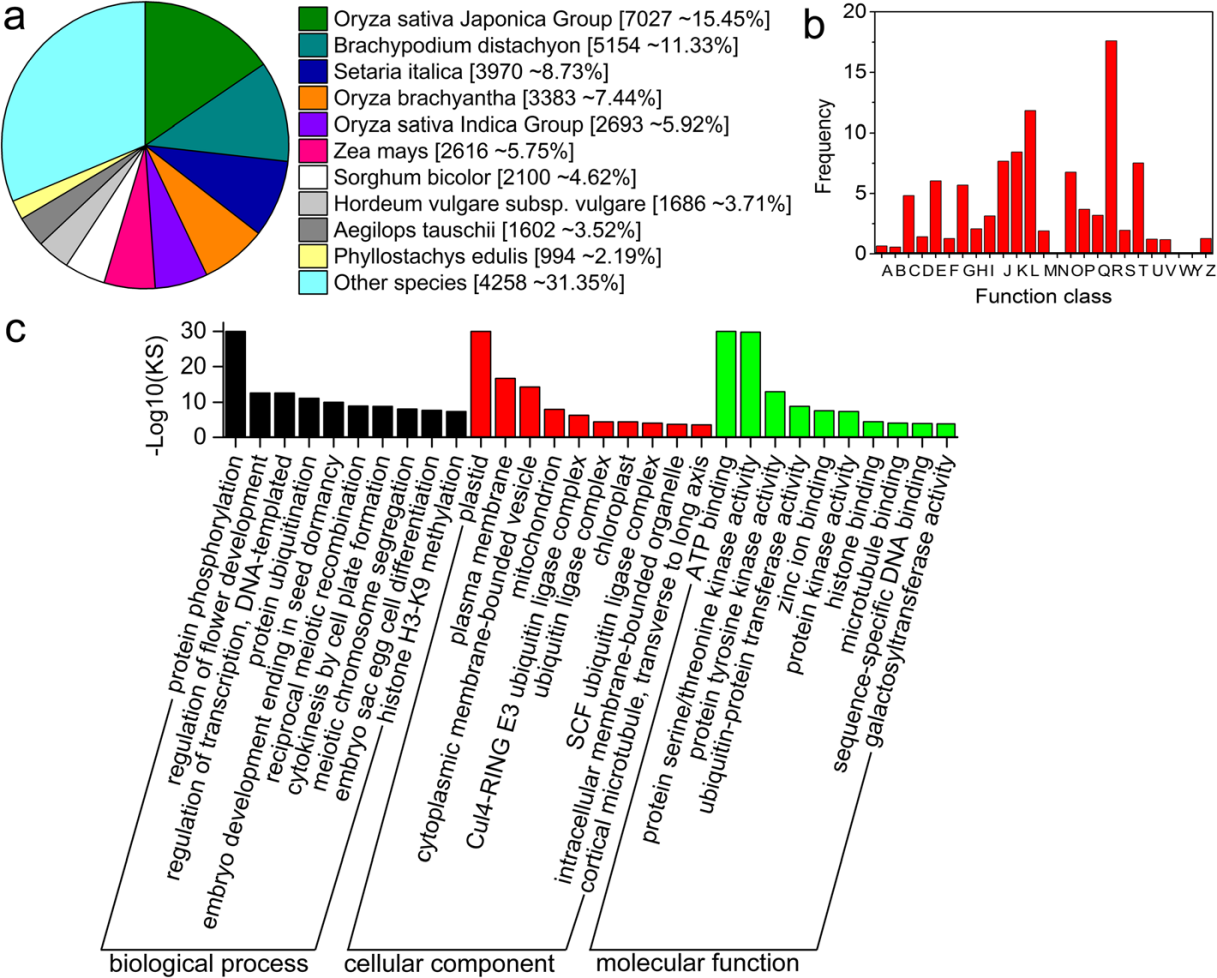
Function annotation and classification of unigenes identified from the transcriptome of *F. macclureana*. (**a**) Nr annotation. (**b**) Clusters of orthologous groups (COG) annotation. Out of 45,516 Nr hits, 13,128 unigenes had a COG classification. A: RNA processing and modification B: Chromatin structure and dynamics C: Energy production and conversion D: Cell cycle control, cell division, chromosome partitioning E: Amino acid transport and metabolism F: Nucleotide transport and metabolism G: Carbohydrate transport and metabolism H: Coenzyme transport and metabolism I: Lipid transport and metabolism J: Translation, ribosomal structure and biogenesis K: Transcription L: Replication, recombination and repair M: Cell wall/membrane/envelope biogenesis N: Cell mobility O: Posttranslational modification, protein turnover, chaperones P: Inorganic ion transport and metabolism Q: Secondary metabolites biosynthesis, transport and metabolism R: General function prediction only S: Function unknown T: Signal transduction mechanism U: Intracellular trafficking, secretion, and vesicular transport V: Defense mechanisms W: Extracellular structures Y: Nuclear structure Z: Cytoskeleton. (**c**) GO annotation. Results were summarized in three main categories: biological process, cellular component and molecular function. The right and left y-axes indicated the number and percentage of unigenes in a certain category, respectively

A total of 47,306 unigenes were annotated (Additional file 2: Table S2). Of these, 45,516 (96.22%) unigenes were found to encode products that showed significant similarity to characterized proteins in the non-redundant protein sequence database (Nr) at an E-value threshold of 10^− 5^ (Table 2). We also found that 7027 (15.45%) unigenes showed similarity to genes found in rice, 11.33% were similar to those found in *Brachypodium distachyon*, and we also found a significant proportion of the unigenes that were similar to those found in *Setaria italica*, *Oryza brachyantha*, and *Zea mays* (Fig. 2a). We identified 24,847 (52.52%), 28,317 (59.86%) and 43,909 (92.82%) unigenes that showed significant matches to entries in the Swiss-Prot, Pfam, and eggnog databases, respectively (Table 2). Many unigenes expressed in the *F. macclureana* transcriptome were functionally annotated as regulators of plant responses to evolutionarily important phenotypes, including membrane stabilization, heat stress response and pathogen defense (Additional file 2: Table S2).

**Table 2:** Statistics of annotation analysis of unigenes

Anno_Database	Annotated_Number	percentage	300 < =length < 1000	length > =1000
COG_Annotation	13,128	27.75	3261	7515
GO_Annotation	34,055	71.99	10,659	17,855
KEGG_Annotation	14,307	30.24	4550	7397
KOG_Annotation	23,492	49.66	6863	12,779
Pfam_Annotation	28,317	59.86	7823	16,896
Swissprot_Annotation	24,847	52.52	7450	14,500
eggNOG_Annotation	43,909	92.82	14,040	21,568
Nr_Annotation	45,516	96.22	15,031	22,271
All_Annotated	47,306	100.00	15,602	22,437

### Functional annotation indicated that many unigenes were involved in metabolism and genetic information processing

We were able to annotate 13,128 unigenes (27.75% of the total) in 25 different categories of the COG (clusters of orthologous groups) classification database (Fig. 2b). Of these, the cluster for “General function prediction only” (3277, representing 24.96% of the 13,128 unigenes annotated by this database) was the largest group, followed by “Replication, recombination and repair” (2202, 16.77%), “Transcription” (1571, 11.97%), and “Translation, ribosomal structure and biogenesis” (1429, 10.88%). The “Signal transduction mechanisms”, “posttranslational modification, protein turnover, chaperones”, “carbohydrate and amino acid transport and metabolism” and “transport and metabolism” categories also contained a significant proportion of the annotated unigenes.

GO enrichment analysis indicated that these predicted unigenes were categorized into three main categories—i.e. biological process (BP), cellular component (CC), and molecular function (MF). As shown in Fig. 2c, for unigenes that were enriched in the BP category, they were mainly involved in biological processes related to reproduction, posttranslational modification and signal transduction; as for those in the CC category, they were mainly involved in cellular components related to membrane, ubiquitin ligase complex, mitochondrion, chloroplast and etc.; while for those in the MF category, they were mainly involved in molecular functions related to signaling transduction (e.g. “ATP binding”, “zinc ion binding”, “protein kinase activity”, and etc.) (Additional file 3: Table S3).

**Fig. 3:**
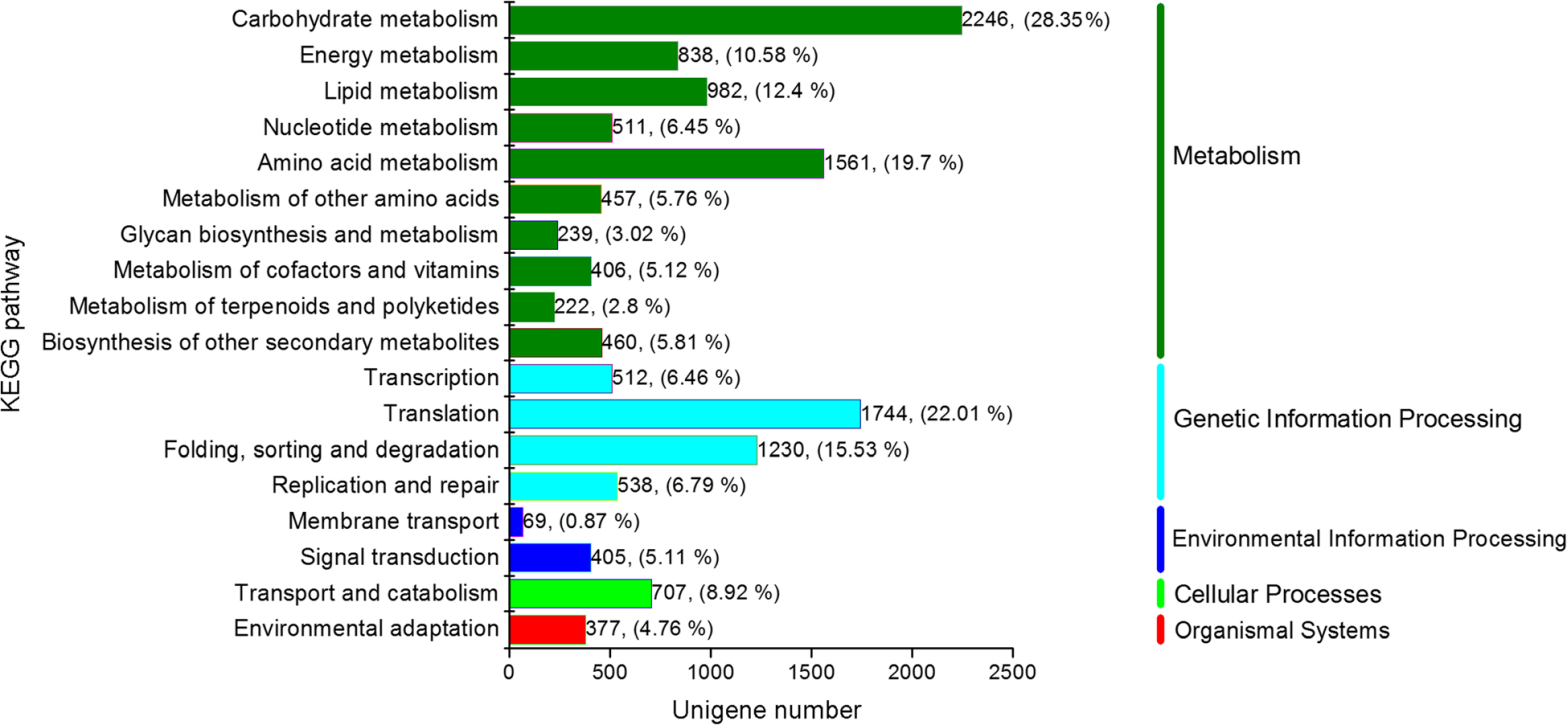
KEGG annotation of unigenes in the transcriptome of *F. macclureana*. The x-axis indicated the number of unigenes in a certain category. The right y-axis showed the main clusters of KEGG pathways

We also mapped 14,307 unigenes (representing 30.24% of the total) to six different KEGG subsystems, including metabolism, genetic information processing, environmental information processing, cellular processes, and organismal systems. As shown in Fig. 3, the majority of these unigenes (7922, representing 66.17% of the 14,307 unigenes classified using KEGG annotations) were assigned to metabolic pathways, including carbohydrate metabolism, energy metabolism, and others. In addition, 4024 unigenes (28.13%) were assigned to genetic information processing, including transcription, translation, and folding, and 474 unigenes (3.31%) were found to be related to membrane transport and signal transduction. We also found 707 genes (4.94%) that were related to transport and catabolism and 377 genes (2.64%) related to environmental adaptation.

### Most BEUs were involved in genetic information processing, environmental adaptation and signal transduction

**Fig. 4:**
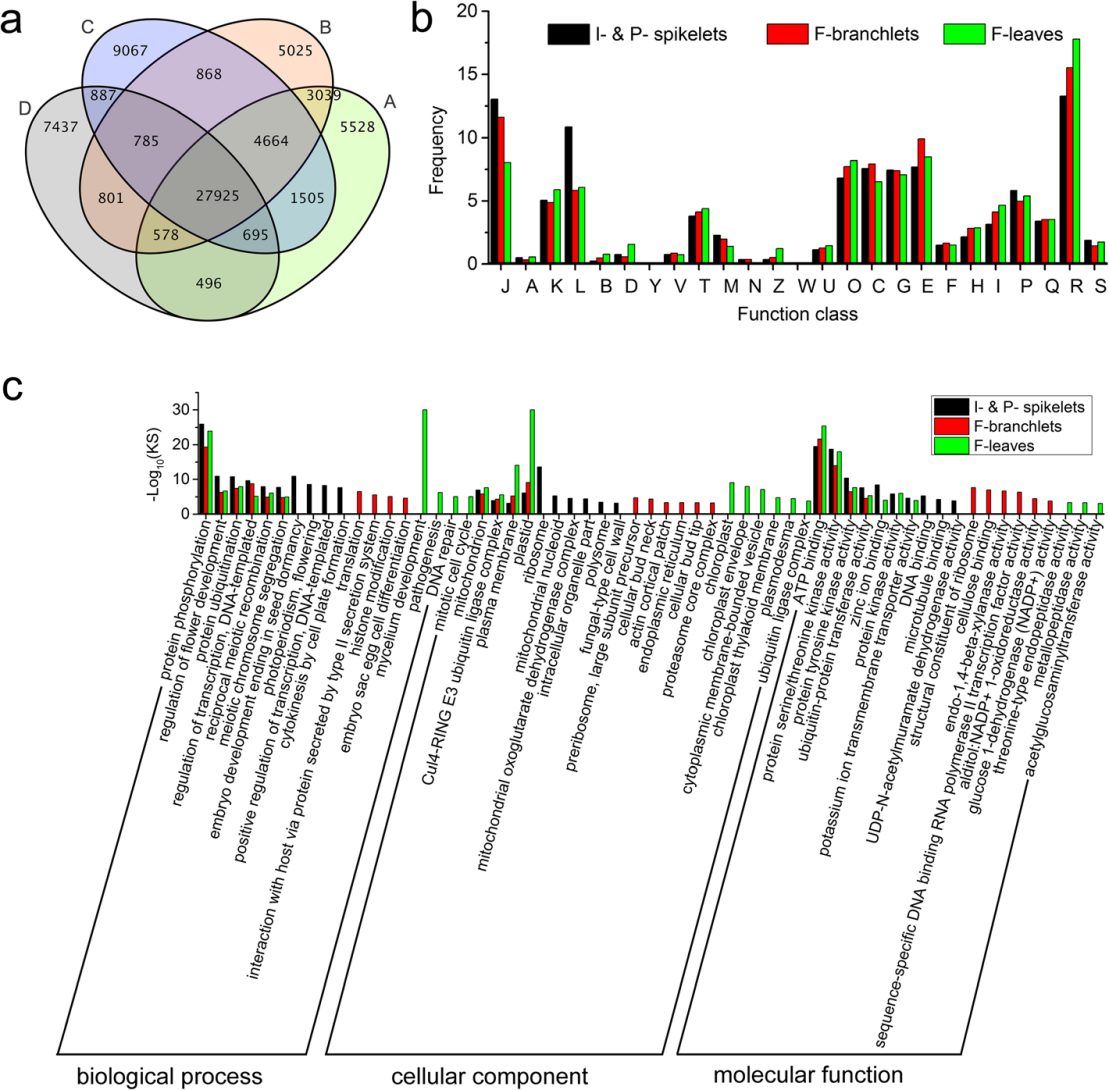
Unigenes that were specifically expressed in different tissues collected from flowering plants of *F. macclureana*. (**a**) Venn diagram of unigenes expressed in spikelets in the initial flower stage (I-spikelets, A) and the peak flower stage (P-spikelets, B), branchlets (F-branchlets, C) and leaves (F-leaves, D) of flowering plants. (**b**) COG annotation of unigenes that were specifically expressed in I-spikelets, P-spikelets, F-branchlets and F-leaves. (**c**) GO enrichment of unigenes that were specifically expressed in I- & P- spikelets, F-branchlets and F-leaves. BP: biological process; CC: cellular component; MF: molecular function

As shown in the Venn diagram (Fig. 4a), we found nearly equal numbers of unigenes that were broadly and specifically expressed in I-spikelets, P-spikelets, F-branchlets, and F-leaves. COG analysis indicated that most BEUs were clustered in signal transduction mechanisms (T), replication, recombination and repair (L), and transcription (K), besides general function prediction only (R). GO enrichment analysis for these BEUs indicated that they were also mainly involved in reproduction, environmental adaptation and signal transduction, which was largely similar with that for all predicted unigenes (Additional file 4: Table S4-a).

KEGG enrichment analysis also indicated that these BEUs were mainly enriched in pathways related to environmental adaptation (including circadian rhythm, endocytosis, and plant-pathogen interactions), signal transduction (including plant hormone signal transduction, phosphatidylinositol signaling system, and inositol phosphate metabolism) and genetic information processing (including spliceosome, mRNA surveillance, and RNA transport and degradation; Additional file 4: Table S4-b).

### The SEUs were mostly involved in carbohydrate metabolism, energy metabolism, and environmental adaptation

As shown in Fig. 4a, we identified 10,653 unigenes that were specifically expressed in spikelets, including 5528 and 5025 unigenes in I- and P-spikelets, respectively. We also found 9067 and 7437 unigenes that were specifically expressed in F-branchlets and F-leaves, respectively. COG annotation indicated that the distribution patterns of SEUs among the 26 terms were similar, with the number of SEUs within each term varying among the three tissues (Fig. 4b).

The GO enrichment analysis indicated that these SEUs not only shared some common GO terms, but also had some particular ones. As shown in Fig. 4c and Additional file 4: Table S4-c, for those SEUs that were enriched in the BP category, they were broadly involved in several important biological processes, including “protein phosphorylation”, “regulation of flower development”, “protein ubiquitination”, “regulation of transcription, DNA-templated”, “reciprocal meiotic recombination” and “meiotic chromosome segregation”. In addition, SEUs in I- and P- spikelets were also involved in some processes related to reproduction; and those in F-branchlets were mainly involved in processes related to posttranslational modification; while those in F-leaves were mainly involved in processes related to plant-pathogen interaction. As for those in the CC category, they were broadly involved in several important cellular components, including “mitochondrion”, “plasma membrane” and “plastid”. In addition, SEUs in I- and P-spikelets were also involved in ribosome and mitochondria; and those in F-branchlets were mainly involved in endoplasmic reticulum and proteasome; and those in F-leaves were mostly involved in chloroplast. As for those in the MF category, they were broadly involved in several molecular functions, including “ATP binding”, “ubiquitin-protein transferase activity” and “protein tyrosine kinase activity”. In addition, SEUs in I- and P-spikelets were also involved in DNA and microtubule binding; those in F-branchlets were also enriched in oxidoreductases activities; and those in F-leaves were also enriched in enzymes involved in carbohydrate metabolism.

As shown in Additional file 5: Figure S1, KEGG pathway analysis indicated that SEUs in I- and F-spikelets mainly mapped to the ribosome pathway, with those in F-branchlets mainly mapped to the ribosome, amino acid biosynthesis, and carbon metabolism pathways, and those in F-leaves mainly mapped to KEGG pathways related to energy metabolism (including oxidative phosphorylation, fatty acid metabolism, and photosynthesis), environmental adaptation (e.g. proteasomes), genetic information processing, and various unrelated metabolic pathways (e.g. tryptophan metabolism, beta-alanine metabolism, and N-glycan biosynthesis).

### DEUs were mostly involved in carbohydrate and energy metabolism, signal transition and environmental adaptation

As shown in Table 3, many unigenes showed differential expressions across all 15 groups sampled. The number of DEUs in each sample pair ranged from 970 between I- vs P-spikelets to 13,577 in NF-leaves vs I-spikelets. For most pairwise comparisons, the number of up- and down-regulated DEUs was approximately the same, except for four groups, including I- vs P-spikelets, F-branchlets vs both I- and P- spikelets, and F-leaves vs P-spikelets.

**Table 3:** Differentially expressed unigenes (DEUs; Fold change >2; FDR < 0.01) among tissues of *F. macclureana*. DEUs_total: the total number of DEUs; DEUs_up (%): the number (and percentage) of up-regulated DEUs; DEUs_down (%): the number (and percentage) of down-regulated DEUs)

Number	Group	DEUs_total	DEUs_up (%)	DEUs_down (%)
1	I-spikelets vs P-spikelets	970	916 (94.43)	54 (5.57)
2	F-branchlets vs I-spikelets	4970	3046 (61.29)	1924 (38.71)
3	F-branchlets vs P-spikelets	5124	3338 (65.14)	1786 (34.86)
4	F-branchlets vs F-leaves	8467	3967 (46.85)	4500 (53.15)
5	F-leaves vs I-spikelets	12,829	6791 (52.93)	6038 (47.07)
6	F-leaves vs P-spikelets	10,791	6625 (61.39)	4166 (38.61)
7	NF-branchlets vs I-spikelets	11,628	6135 (52.76)	5493 (47.24)
8	NF-branchlets vs P-spikelets	10,809	5893 (54.52)	4916 (45.48)
9	NF-branchlets vs F-branchlets	6524	3275 (50.20)	3249 (49.80)
10	NF-branchlets vs F-leaves	11,670	5902 (50.57)	5768 (49.43)
11	NF-branchlets vs NF-leaves	3853	1946 (50.51)	1907 (49.49)
12	NF-leaves vs I-spikelets	13,577	6921 (50.98)	6656 (49.02)
13	NF-leaves vs P-spikelets	11,718	6130 (52.31)	5588 (47.69)
14	NF-leaves vs F-branchlets	11,659	5606 (48.08)	6053 (51.92)
15	NF-leaves vs F-leaves	5032	2492 (49.52)	2540 (50.48)

**Fig. 5:**
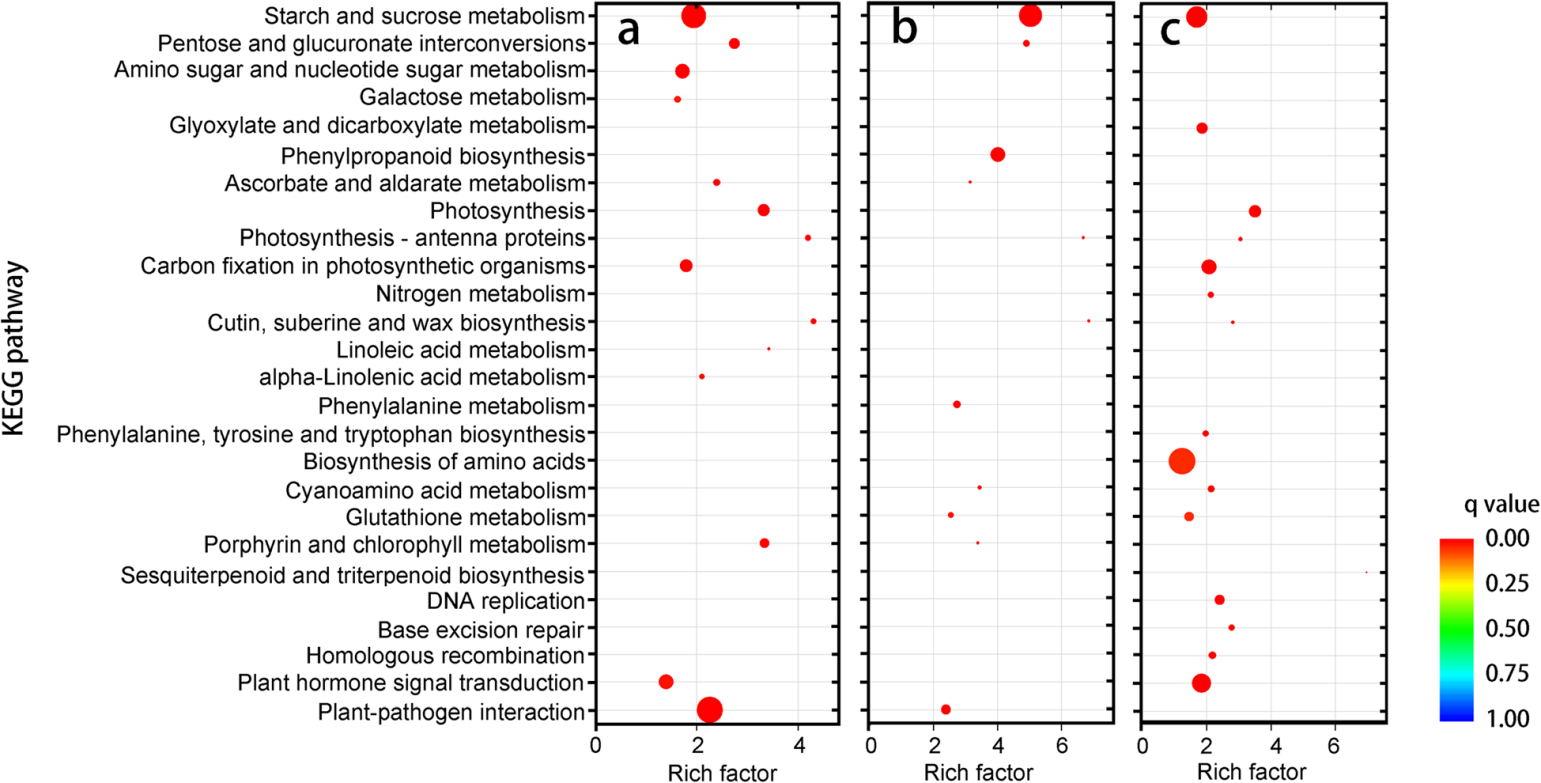
KEGG annotation of unigenes that were specifically expressed in P-spikelets (**a**), F-branchlets (**b**) and F-leaves (**c**) of arrow bamboo flowering plants. The size of dots is proportional to the number of unigenes

The Venn diagram of DEU sets shows that 5494 unigenes were differentially expressed in F-branchlets/F-leaves vs I- and P-spikelets. For those DEUs that were up-regulated in spikelets, they are mainly mapped to KEGG pathways related to carbohydrate metabolism, plant-pathogen interactions and DNA repair (Fig. 5a). Notably, among the 970 DEUs identified between I- and P-spikelets, 916 up-regulated DEUs were mapped to KEGG pathways related to metabolic activity (Additional file 6: Table S5).

A total of 5494 unigenes were differentially expressed in the DEU sets of spikelets/F-leaves vs F- branchlets. Upregulated DEUs in F-branchlets were mapped to KEGG pathways including phenylalanine metabolism, phenylpropanoid biosynthesis, ABC transporters, and flavone and flavonol biosynthesis (Fig. 5b). Those that were upregulated in F- and NF-leaves vs F- branchlets were mainly mapped to plant hormone signal transduction, homologous recombination, base excision repair, and mismatch repair (Additional file 6: Table S5). Notably, 3275 (50.20% of the total) DEUs found between NF- and F-branchlets were upregulated; these were mainly mapped to KEGG pathways related to replication and recombination (Additional file 6: Table S5). Those that were downregulated were mainly mapped to carbon fixation and photosynthesis (Additional file 6: Table S5).

We also found that 6966 (43.69% of the total) DEUs found in spikelets/F-branchlets vs F-leaves were up-regulated, and were mainly mapped to KEGG pathways related to carbohydrate metabolism (Fig. 5c). 2492 (49.52%) DEUs in NF-vs F-leaves were up-regulated, and these were mainly mapped to starch and sucrose metabolism (Additional file 6: Table S5). In contrast, downregulated DEUs were mainly mapped to KEGG pathways related to photosynthesis (Additional file 6: Table S5).

### Two putative *FT* orthologs were regulated differently in the circadian rhythm–plant pathway

Among the 5032 DEUs identified between NF- and F-leaves, 70 were mapped to the circadian rhythm–plant KEGG pathway (Additional file 7: Figure S2) and 10 of them showed differential expressions (Additional file 8: Table S6). Notably, c109220.graph_c0 and c110963.graph_c4 were both annotated as *FT* orthologs: the former was a putative bamboo ortholog of *Heading date 3a* (Swissprot: PE = 1 SV = 1), and the latter was another ortholog of rice *FT*; we designated them as *FmHd3a* and *FmFT*, respectively.

**Fig. 6:**
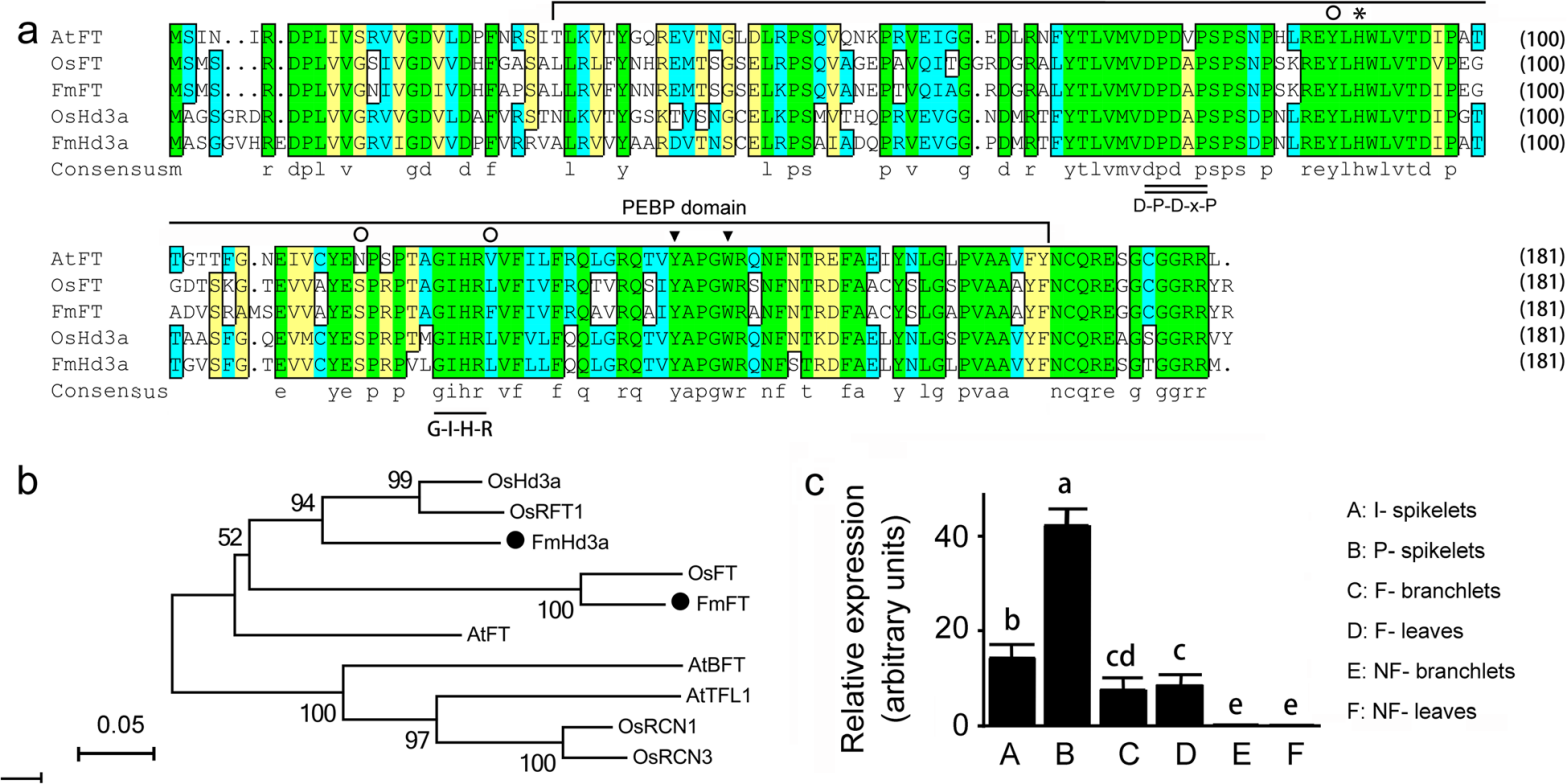
Alignment (**a**) and phylogenetic analyses (**b**) of FT/TFL1 proteins from *F. macclureana* and other plants, as well as the expression pattern of *FmHd3a* across six different tissues collected by RT-qPCR (**c**). The involving sequences and their accession numbers are: three FT/TERMINAL FLOWER 1 (TFL1) proteins from *A. thaliana*: AtFT (AT1G65480.1), AtTFL1 (NP_196004.1) and BROTHER OF FT AND TFL1 (AtBFT, AT5G62040.1); and four FT/TFL1 proteins from *Oryza sativa*: OsFT (XP_015619436.1), OsRFT1 (rice RICE FLOWERING LOCUS T 1 (RFT1), BAO03220.1), OsHd3a (XP_015611892.1) and two RCNs (putative TFL1/CENTRORADIALIS (CEN) orthologs): OsRCN1 (AAD42895.1) and OsRCN3 (AAD42896.1). Amino acid sequences with double underline and single underline indicate the critical motifs in PEBP proteins [27]; asterisk and triangles indicate the invariant histidine in the PEBP domain [28] and two conserved sites in inducer FTs [29], respectively. Hollow circles indicate the position where the critical amino acid variation occurred between *Arabidopsis* TFL1 and FT, an activator and a repressor of flowering [30]. Relative expression levels were calculated using the 2^−ΔΔCT^ method to reflect expressions relatively more veritably. The statistical significance was tested by one-way ANOVA, considering *P* < 0.05 and *P* < 0.01 as statistically significant and extremely significant, respectively. Significant differences in transcript abundance between different tissues were then compared by Duncan’s multiple range test using SPSS 17.0 (SPSS Inc., Chicago, USA)

As shown in Fig. 6a, protein sequence alignment indicated that both FmFT and FmHd3a had high amino acid sequence similarities (77.14%) with the known FT/TFL1 proteins and had the critical amino acids of FT/Hd3a proteins. For example, they both carry a conserved phosphatidylethanolamine-binding protein (PEBP) domain, D-P-D-x-P and G-x-H-R motifs, and the invariant histidine (asterisk), all of which are relevant to the ability of PEBP proteins to bind phosphoryl ligands, hence interfering with certain kinases and effectors [27, 28]. Furthermore, all five proteins carry tyrosine-139 and tryptophan-143 (triangles) in the PEBP domain, two conserved sites in FT homologs acting as flowering inducers [29]. Whereas, our comparison also revealed that there were differences in amino acid sequences between FTs and Hd3as.

Additionally, phylogenetic analysis indicated that these ten proteins were subdivided into two distinct subgroups (Fig. 6b). FT and Hd3a proteins from *O. sativa* (OsFT, OsRFT1 and OsHd3a), *F. macclureana* (FmFT and FmHd3a) and *A. thaliana* (AtFT) were clustered in a branch. Furthermore, OsFT and FmFT were clustered together, just like OsHd3a and FmHd3a; they were both closer to each other than they were to AtFT.

Interestingly, two FT orthologs were regulated differently in NF- vs F- leaves. *FmHd3a* was significantly upregulated in F-leaves (FDR = 4.23, log_2_FC = 5.55), while *FmFT* was significantly downregulated in F-leaves (FDR = 4.25E-07, log_2_FC = − 4.81). RT-qPCR analysis also showed that *FmHd3a* was significantly more highly expressed in I- /P- spikelets and F-leaves than in NF-leaves or NF- branchlets (Fig. 6c).

### WGCNA results identified gene modules related to specific tissues

WGCNA results showed that unigenes expressed in the six different tissues of flowering and nonflowering plants tested here clustered into 18 branches representing 18 different genetic modules (Additional file 9: Figure S3a). Unigenes within each module were highly co-expressed, while those in different modules were co-expressed to a lower degree (Additional file 9: Figure S3b). In six of the samples collected, we identified nine significant gene modules including 1344 unigenes. Here, correlation coefficient of a module with a related trait >0.7 was used as a threshold of significance (Additional file 9: Figure S3c). Notably, these six tissues were more strongly divided into clades according to whether they were flowered or not rather than by the differences among tissues (Additional file 9: Figure S3d).

In addition, the unigenes in gene modules relating to I - and P- spikelets were most strongly enriched in KEGG pathways related to carbohydrate metabolism, genetic information processing, and environmental information processing. In contrast, those related to F- and NF- branchlets were mostly enriched in KEGG pathways related to metabolism, plant hormone signal transduction, and genetic information processing. The gene modules related to F-leaves were enriched in pathways related to plant hormone signal transduction and protein processing, while the gene modules related to NF-leaves were enriched in KEGG pathways related to oxidative phosphorylation (Additional file 10: Table S7).

### Identification of SSRs

**Fig. 7:**
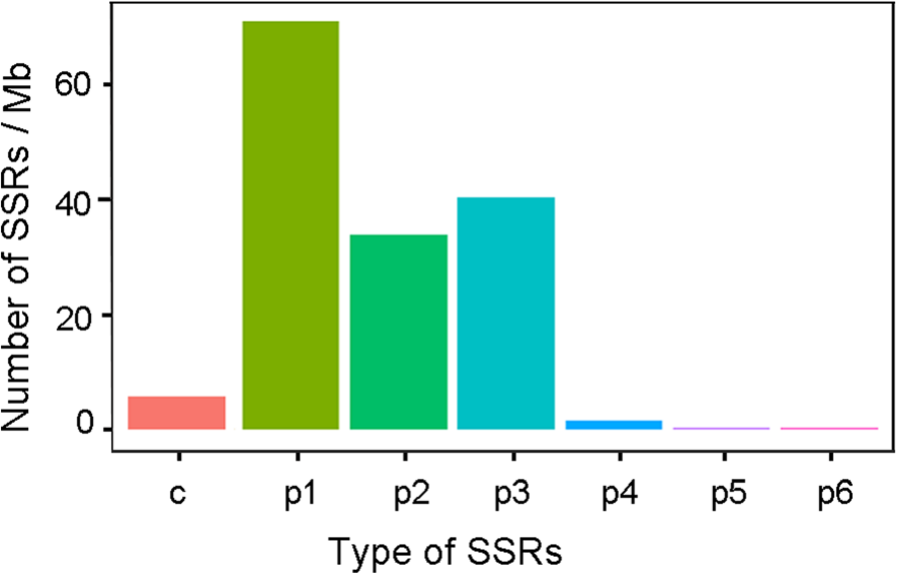
Densities of different SSR types. c and p1–6 represent mono-, di-, tri-, tetrad-, penta- and hexa-nucleotide repeats, respectively

We detected a total of 9296 SSRs in 7668 unigenes longer than 1000 bp (Additional file 11: Table S8). 1628 (21.23%) unigenes contained more than one SSR. Mono-nucleotide repeats were the most common (46.28% of all SSRs) at a density of 71 SSRs per Mb, followed by tri- (26.32%) and di- (22.06%) nucleotide repeats, with densities of 40 and 32 SSRs per Mb, respectively (Fig. 7).

## Discussion

### Activated *Hd3a* expression probably accelerates flowering in *F. macclureana*

FT is a key floral regulator that controls the timing of flowering and seasonal growth cessation in response to light and the circadian clock in many plant species [8, 10, 31]. In this study, *FmHd3a*, a bamboo ortholog of rice *FT*, was significantly expressed only in tissue samples collected from flowering plants. In rice, *Hd3a* functions as a major photoperiodic flowering regulator and participates in the *OsGI*–*Hd1*–*Hd3a* module, which is similar to the *GI*-*CO*-*FT* module in *Arabidopsis* [32]. *Hd1* activates and suppresses *Hd3a* expression by promoting heading under the short day (SD) and long day (LD) conditions, respectively [33, 34]. As *F. macclureana* rarely blossoms on the QTP, which experiences a long photoperiod with a low ratio of red to far-red light, it may have evolved specific reproductive strategies involving flowering-related pathways in response to photoperiodic cues to ensure long vegetation growing period. It is probably that the weak light intensity with a low proportion of blue light might activate *Hd3a* expression even in the LD conditions, thereby accelerating flowering. Notably, reproduction pathways play an important role in the mechanisms of plant adaptation to extreme environments. Previous studies showed that the phytochrome and flowering time regulatory protein 1 (PFT1) from *Crucihimalaya himalaica*, a close relative of *Arabidopsis* and *Capsella*, grows on the QTP, showed signs of positive selection for adaptive divergence [35]. We presume that the function of *Hd3a* in promoting flowering is likely to be conserved between bamboo and rice, because both of them belong to the Poaceae.

Interestingly, *FmFT* and *FmHd3a* were regulated differently in NF- vs F- leaves, although they were both annotated as FT regulators in the circadian rhythm-plant pathway. Basically, FT sub-family protein acts as florigen; the diverse functions of the *FT* gene family in flowering regulation had been demonstrated in many different plant species [29]. For example, sugar beet BvFT1 repressed flowering and the divergence within three amino acids of an external loop in PEBP domain was demonstrated to be the major cause [36]. Similarly, a single amino acid exchange in PEBP domain was sufficient to convert FT to TFL1, an activator and a repressor [30, 37]. In our study, five proteins carry invariant amino acids in positions where variation occurred; however, there were many differences in predicted amino acid sequences between them in PEBP domain. Especially for some positions, amino acids of AtFT and FmHd3a are the same, but different from that of FmFT (Fig. 6a). Possibly, FmFT acts as a repressor FT due to the conversion of certain amino acids in PEBP domain, while FmHd3a acts as an inducer one. Thus, we suspect that the floral transition of *F. macclureana* is regulated by a complex regulatory network in which at least two unique *FT* orthologs interact with the circadian clock pathways. However, how these circadian clock pathways mediate the activation of *FmHd3a* and *FmFT* in response to light signalling remains to be elucidated by future research.

Notably, we detected *FmHd3a* expressions in all four tissues collected from the flowering plants, but not in NF-leaves or NF-branchlets. We also noticed that all plants were sampled from the same provenance and grown in chambers under the same conditions after transplantation, but all non-flowering plants each had a longer section of rhizome than the flowering ones. Given that rhizome can provide nutrients for plants to maintain normal growth, thus, we hypothesize that the broken balance between vegetative and reproductive growth after transplantation result in the floral transition. Additionally, the DEUs upregulated in F-leaves relative to NF-leaves were mainly enriched in KEGG pathways related to starch, sucrose, and galactose metabolism, and corresponding down-regulated DEUs were mapped to the light and carbon fixation, plant circadian rhythm, and photosynthesis pathways. Therefore, we speculate that bamboo *FT* orthologs might be regulated by upstream regulators involved in carbohydrate metabolism.

### Carbohydrate metabolism and signal transport may be major factors in floral transition, organogenesis, and death after flowering

Bamboo exhibits excellent flexibility and fracture toughness, and so far, the presence of fibers within the bamboo culm was thought responsible for these remarkable mechanical properties [38]. And the development of plant fibers is accompanied by the of carbohydrate metabolism [39]. Perhaps this is the reason why many DEUs were involved in carbohydrate metabolism pathway. Interestingly, our results indicated that starch and sucrose metabolism was a major enriched KEGG pathway for the DEUs from several combination pairs, including NF- vs F-leaves, branchlets/leaves vs I- & P- spikelets. Transcripts and metabolic signatures of maize leaves have shown that the balance between transitory starch and sucrose is associated with the autonomous floral transition [40]. And in *Lilium,* carbohydrates have been found to be transported from the vascular bundles to floral organs during reproduction [41]. Yang et al., (2017) also reported that the deficiency in the resources in male flowers reduced pollen viability in *Tapiscia sinensis* due to biased carbohydrate transport toward the female flowers [42]. Therefore, we suspect there may be a correlation between DEUs related to starch and sucrose metabolism and arrow bamboo floral organ development.

In rice, excessive uridine 5′-diphosphoglucose-glucose (UDPG) can result in programmed cell death, accumulation of reactive oxygen species and an increase in the caspase-like activity [43] and inactivate starch synthase disrupted normal male reproduction by delaying programmed cell death in cotton [44], suggestive of a correlation between starch and sucrose metabolism and death. Bamboo flowering, especially in masting species, often causes plants to wilt and die after setting seed. It is possible that increased starch and sucrose metabolism might trigger the excessive accumulation of reactive oxygen species and result in the altered activity of key enzymes in important biological pathways.

In the present study, unigenes related to the signal transduction pathways were significantly upregulated in the tissues of flowering arrow bamboo plants. The transcriptomic profiles of *Posidonia oceanica* also showed a strong metabolic activation of hormones in the heat stress-induced flowering plants [45]. Signal transduction-related genes were also found to have undergone both significant positive selection and expansion events on the adaptive evolution of *Crucihimalaya himalaica* [35] and *cyanobacterium Trichormus* sp. NMC-1 [46] on the QTP. In the present study, unigenes related to the signal transduction pathways were significantly upregulated in the tissues of flowering arrow bamboo plants. We suspect that this may be due to the long distance transport of the FT proteins, which ensures floral promotion at the shoot apex [47], or the phytohormone signaling and calcium signaling, which play diverse roles in the specification of flower organs during arrow bamboo reproductive development [48, 49].

### *F. macclureana* has presumably evolved an integrated mechanism to adapt to the harsh environment of the QTP

We detected the broad expressions of unigenes encoding putative Hsp proteins, such as *heat shock protein 70* (*Hsp70*) and *heat shock protein 90* (*Hsp90*). Both Hsp70 and Hsp90 are important for maintaining cellular protein homeostasis under stress conditions and they function by activating other targets [50–52]. It is likely that the sudden exposure to the higher temperature of the lab (i.e. outside the QTP) triggered the expressions of these unigenes. Warmer temperatures can greatly reduce flowering synchrony among individuals from 72 woody and herbaceous species [53]. It was also reported that *P. oceanica*, a highly clonal and long-lived species, massively bloomed after a simulated heatwave [45]. Given the cold temperatures present at the high-altitude regions of the QTP [26], it is reasonable to presume that *F. macclureana* has developed into a heat-sensitive but not heat-tolerant bamboo species and flowering is probably a stress-induced response to the higher temperature in the lab.

DNA repair and disease-resistance pathways have been found to play a crucial role in the highland adaptation of Tibetan highland plants [46, 54]. In the present study, we detected significant expressions of many unigenes related to pathogen response that contained either a nucleotide binding (NB)-ARC domain or a leucine-rich repeat (LRR) domain, which were present in most resistance (R) proteins [55–57]. We also identified many differentially expressed unigenes that were significantly enriched in the DNA repair pathways. Since relatively fewer species of pathogenic microorganisms and intense UV radiation exist on the QTP [58], it is reasonable to presume that *F. macclureana* has evolved a relatively narrow range of pathogen specificity and specific DNA-repair mechanisms. Sudden exposure to the lab environment, which contains a heavier load of pathogens and weak light intensity, may have induced an innate defensive response of *F. macclureana*, and those that were enriched in the plant-pathogen interaction and DNA repair response pathways may be important for *F. macclureana* to cope with the new environment present in the lab. Although further studies are needed to investigate the molecular mechanisms responsible for the putative adaptive evolutionary changes, this study provides insights into how plants adapt to harsh and extreme environments.

## Conclusions

In the present study, we constructed a novel de novo transcriptome analysis for *F. macclureana*. Based on two major KEGG pathways of carbohydrate metabolism and signal transduction that DEUs were enriched in, as well as the different regulation of *FmFT* and *FmHd3a* in NF- vs F- leaves, we speculate that both environmental signals and physiological status have effect on floral transition in *F. macclureana*. Significant expressions of unigenes enriched in DNA repair and plant-pathogen interaction pathways may reflect the adaptation of *F. macclureana* to its high radiation and pathogen-specific environment on the QTP. We identified both similarities and differences in adaptive mechanisms (e.g., disease-resistance and DNA repair pathways) and stress-induced flowering mechanisms (e.g., carbohydrate metabolism and signal transduction pathways) among plants that grow at high altitudes. Although further experimental verification is needed, our results provide insight into the regulation of flowering time in highland bamboo as well as how this species adapts to harsh and extreme environments.

## Methods

### Tissues collection

The studied plant species is highland arrow bamboo (*Fargesia macclureana*), and it grows mainly as an underbrush of coniferous forest or coniferous and broad-leaved mixed forest, and sometimes forms a pure population in the QTP at an altitude of approximately 2000 ~ 3800 m above sea level (Fig. 1). *F. macclureana* was formally identified by Stapleton in 1993 [25] and detailed explanations are provided in the volume 22 of Flora of China (http://foc.iplant.cn/) [59]. A voucher specimen of this material has been deposited in the Bamboo Research Institute of Nanjing Forestry University. A total of six seedlings of *F. macclureana* were sampled from the wild with the permission of the local forestry department and collected from the Bayi District, Linzhi City, Tibet, China (29°46′ 0.95″ N, 94 44′46.36″ E, altitude: ~ 2200 m). They were dig out of the ground, each with a section of rhizome, which can provide nutrients for plants. Then they were all transferred to individual pots at the State Forestry Administration Key Open Laboratory at the International Center for Bamboo and Rattan in Beijing (N: 39°59′ 17.52″, E: 116 28′46.06″, altitude ~ 34 m). During growth, the plants were maintained at 28 ± 1 °C and 50–55% relative humidity under a 16/8 h (light/dark) photoperiod regimen with a light intensity of 200 μmol · m^− 2^ · s^− 1^. All the seedlings were watered with a 1/3 B5 macronutrient nutrient solution three times a week. After 20 days, four seedlings which had shorter rhizomes flowered, while the other two didn’t blossom until the time of sampling. One month later, we collected samples of six tissues for further de novo sequencing, including inflorescences in the initial flower stage (I-spikelets), inflorescences at the peak flower stage (P-spikelets), branchlets of the flowering plants (F-branchlets), leaves of the flowering plants (F-leaves), branchlets of the non-flowering plants (NF-branchlets) and leaves of the non-flowering plants (NF-leaves). We collected three independent replicates of each tissue type.

### RNA extraction, quantification, and qualification

Total RNA was extracted from each of the six unique tissues mentioned above using a RNeasy plant RNA extraction kit (Qiagen, Dusseldorf, Germany), and the extraction procedure was performed according to the manufacturer’s instructions. RNA degradation and contamination were monitored using 1% agarose gels. RNA purity was checked using a NanoPhotometer® spectrophotometer (Implen GmbH, Munich, Germany). RNA concentration was measured using a Qubit® RNA Assay Kit and a Qubit®2.0 Fluorometer (Life Technologies, CA, USA). RNA integrity was assessed using an RNA Nano 6000 Assay Kit run on an Agilent Bioanalyzer 2100 system (Agilent Technologies, CA, USA).

### Library preparation for transcriptome sequencing

Library construction and RNA-Seq were performed by the Biomarker Biotechnology Corporation (Beijing, China). A total of 3 μg RNA per sample was used for RNA preparation. Briefly, mRNA was purified from total RNA using poly-T oligo-attached magnetic beads, followed by fragmentation carried out using divalent cations at elevated temperature in NEBNext First Strand Synthesis Reaction Buffer (5×). First strand cDNA was synthesized using random hexamer primers and M-MuLV Reverse Transcriptase (RNase H-). Second strand cDNA synthesis was subsequently performed using DNA Polymerase I and RNase H. The remaining overhangs were converted into blunt ends via the exonuclease and polymerase activities. Next, the 3′ ends of the DNA fragments were adenylated and ligated to the NEBNext adaptors with hairpin loop structures to prepare samples for hybridization, this was to select cDNA fragments that are 150–200 bp in length. Library fragments were then purified using an Agencourt AMPure XP system (Beckman Coulter, Brea, CA, USA), and 3 μl USER enzyme (New England Biolabs, Ipswich, MA, USA) was added to the size-selected, adaptor-ligated cDNA at 37 °C for 15 min followed by 5 min at 95 °C before PCR. PCR was performed using Phusion High-Fidelity DNA polymerase (Thermo Fisher, Waltham, MA, USA), universal PCR primers, and the Index (X) Primer. Finally, the PCR products were purified using the AMPure XP system and library quality was assessed on the Agilent Bioanalyzer 2100.

### Clustering and sequencing

The clustering of index-coded samples was performed using a cBot Cluster Generation System and a TruSeq PE Cluster Kit v3-cBot-HS (Illumina, San Diego, CA, USA), and all experimental procedures were performed according to the manufacturer’s instructions. After that, library preparations were sequenced on an Illumina Hiseq 2000 platform and paired-end reads were generated.

### De novo transcriptome assembly

Raw data in fastq format were first processed using in-house perl scripts. Clean data were obtained by removing low-quality reads and reads that contain the adapters or poly-N sequences. Meanwhile, we checked the quality of our unassembled read dataset by examining various measures including Q20, Q30, GC-content, and sequence duplication. All the downstream analyses were performed using high-quality clean data.

The transcriptome was assembled using clean reads from all libraries and samples. The assembly was produced using Trinity [60] with min_kmer_cov set to 2 and all other parameters set to their respective default values.

### Functional annotation of the transcriptome

Gene function was annotated using the following databases: Nr (NCBI non-redundant protein sequences), Pfam (Protein family), KOG/COG/eggNOG (Clusters of Orthologous Groups of proteins), Swiss-Prot (a database of manually annotated and reviewed protein sequences), KEGG (the Kyoto Encyclopedia of Genes and Genomes), and the GO (Gene Ontology) database.

### Quantification of gene expression levels

Gene expression levels were estimated using RSEM [61] for each sample: clean data were mapped back onto the assembled transcriptome, followed by a read count for each gene. The expression levels of unigenes were calculated and normalized using FPKM (fragments per kb per million fragments) [62].

### Expression analysis of broadly and specifically expressed unigenes

For all unigenes, those that were expressed in all six tissues were defined as broadly expressed unigenes (BEUs). Similarly, unigenes that were specifically expressed in only one tissue were defined as specifically expressed unigenes (SEUs). The identification of BEUs and SEUs was conducted by using tools on the BMKCloud platform (http://www.biocloud.net).

### Weighted gene co-expression network analysis (WGCNA)

WGCNA was performed on all unigenes identified using the *WCGNA* R package. We calculated the adjacency matrices and performed the topological overlap measures (TOMs), which show the degree of overlap in shared neighbors between pairs of genes in the network to define gene clusters in our transcriptome dataset. 1 − TOM was used as a dissimilarity measure for hierarchical clustering and module detection. Modules of the clustered genes were then selected using the Dynamic Tree Cut algorithm as implemented by WGCNA. To identify modules that are significantly related to particular tissues, expression profiles of each module were summarized by a module eigengene (ME) and the correlations between the modules and corresponding tissues were calculated.

### Expression analysis of differently expressed unigenes (DEUs)

Before analysis, we conducted a principal component analysis (PCA) and removed one replicate that showed an inconsistent expression pattern in the NF-branchlets and NF-leaves to ensure consistency in the expression patterns of unigenes between replicates (Additional file 12: Figure S4).

Expression analysis of the DEUs between pairs of tissues/groups was performed using the *DESeq* package (1.10.1) in R. *DESeq* provides statistical routines for identifying differential expression in the digital gene expression data using a model based on the negative binomial distribution. The resulting *P* values were adjusted using the Benjamini-Hochberg method for controlling the false discovery rate [63]. Here, uni-transcripts with an absolute value of log_2_ ratio ≥ 2, an FDR significance score < 0.01, and an adjusted *P*-value <0.05 were deemed to be differentially expressed.

### Gene ontology (GO) and Kyoto encyclopedia of genes and genomes (KEGG) pathway enrichment analysis

To understand the higher-level functions of the observed unigenes, we performed GO term annotation and KEGG pathway enrichment analysis using BMKCloud (http://www.biocloud.net/ [64];). We used KOBAS 2.0 [65] to test the statistical enrichment of differentially expressed genes in KEGG pathways. Pathways with *P* values <0.05 were considered significantly enriched.

### Protein-protein interactions (PPIs)

The DEU and SEU sequences were queried using BLASTX against the related species to predict PPIs that the DEUs and SEUs may be involved in. This search procedure was capable of identifying PPIs that may be similar to any others found in the STRING database (http://string-db.org/). These PPIs were then visualized using Cytoscape [66].

### Detection of SSRs

Picard-tools version 1.41 and samtools version 0.1.18 were used to sort data, remove duplicated reads, and merge the bam alignment results of each sample. SSRs were identified using MISA (https://webblast.ipk-gatersleben.de/misa/).

### Validation of *FmHd3a* transcript levels by RT-qPCR

To verify the expression pattern of the *FmHd3a*, we used RT-qPCR to assess the expressions of *FmHd3a* in six distinct tissues. First-strand cDNA was synthesized from total RNA extracted by using a reverse transcription system (Promega, Madison, WI, USA) following the manufacturer’s instructions. Each RT-qPCR amplification was performed at least three times, and *NTB* and *TIP41* were used as internal controls [67]. Primers for these genes are listed in Additional file 13: Table S9. The relative expression levels of *FmHd3a* in different tissues were calculated using the 2^-ΔΔCT^ method [68]. The statistical significance of differences in the mean levels of expression was tested using a one-way ANOVA. Significant differences in transcript abundance between different tissues were then compared using Duncan’s multiple range tests as implemented by SPSS version 17.0 (IBM SPSS, Chicago, USA). We considered mean differences at *P* < 0.05 and *P* < 0.01 to be statistically significant and highly statistically significant, respectively.

## Supplementary information

**Additional file 1: Table S1.** Statistic of sequencing and assembly data.

**Additional file 2: Table S2.** 47,306 unigenes were annotated and their predicted functions.

**Additional file 3: Table S3.** The top10 enriched GO terms in three main categories for all unigenes identified.

**Additional file 4: Table S4.** The most enriched GO terms and KEGG pathways for the specially expressed unigenes (SEUs) and broadly expressed unigenes (BEUs) in all tissues collected from flowering plants.

**Additional file 5: Figure S1**. KEGG annotation of unigenes that were specifically expressed in P-spikelets (**a**), F-branchlets (**b**) and F-leaves (**c**) of arrow bamboo flowering plants. The size of dots is proportional to the number of unigenes.

**Additional file 6: Table S5.** KEGG enrichment of differentially expressed unigenes (DEUs) between different tissues.

**Additional file 7: Figure S2**. Hub unigenes in regulatory networks of flowering identified based on analysis of DEUs among tissues. Unigenes c109220.graph_c0 and c110963.graph_c4, showing differential expressions between NF-leaves and F-leaves, are both bamboo orthologs of *FLOWERING LOCUS T* (*FT*), which was marked with a red square; while unigenes down-regulated were marked with green squares.

**Additional file 8: Table S6.** Expressions of 10 unigenes that were differentially expressed between F-leaves vs NF-leaves and mapping into the circadian phythm-plant KEGG pathway.

**Additional file 9: Figure S3**. Weighted gene co-expression network analysis (WGCNA) of all unigenes identified in the transcriptome of *F. macclureana*. (**a**) The phylogenetic tree diagram and the heat map related to the traits. This diagram is divided into three parts: the cluster tree of gene system, the module color of corresponding genes, and the correlation between genes related to each trait in tested samples and its module. The redder the color, the more positive the correlation; conversely, blue is negatively correlated. (**b**) Gene co-expression network heatmaps drawn by randomly selected 1500 genes, in which the left and the upper sides are the symmetrical system clustering tree of gene network/module, and the lower right area indicates the dissimilarity between genes, and the smaller the value is, the darker the color is. (**c**) Module and trait correlation heat map showing the relationship between a module and a given trait. The closer the correlation between a shape and a module is to the absolute value of 1, it is likely that this trait is related to the module gene work. (**d**) Systematic clustering tree of samples based on unigenes expressions.

**Additional file 10: Table S7.** KEGG enrichment of unigenes in nine significant gene module relating to P-spikelets.

**Additional file 11: Table S8.** 9296 SSRs identified from 7668 unigenes.

**Additional file 12: Figure S4.** Principal component analysis (PCA) of unigenes expressions for 18 samples collected from inflorescences in the initial and peak flower stage (I- and P- spikelets), branchlets and leaves of flowering and non-flowering bamboo plants (F/NF-branchlets and F/NF-leaves).

**Additional file 13: Table S9**. Primer pairs used for RT-qPCR.

## Data Availability

The RNA sequencing dataset generated during the current study have been submitted to NCBI Sequence Read Archive (SRA) database (https://www.ncbi.nlm.nih.gov/sra) with the accession number PRJNA544133.

## Abbreviations

BEUs: Broadly expressed unigenes
BP: Biological process
CC: Cellular component
COG: Clusters of orthologous groups
DEUs: Differentially expressed unigenes
F-branchlets: Branchlets of the flowering plants
F-leaves: Leaves of the flowering plants
FPKM: Fragments per kb per million fragments
GO: Gene ontology
I-spikelets: Inflorescences in the initial flower stage
KEGG: Kyoto Encyclopedia of Genes and Genomes
ME: Module eigengene
MF: Molecular function
NF-branchlets: Branchlets of the non-flowering plants
NF-leaves: Leaves of the non-flowering plants
Nr: NCBI non-redundant protein sequences
PCA: Principal component analysis
Pfam: Protein family
PPIs: Protein-protein interactions
P-spikelets: Inflorescences at the peak flower stage
QTP: Qinghai–Tibet Plateau
SEUs: Specifically expressed unigenes
SSRs: Simple sequence repeats
TOMs: topological overlap measures
WGCNA: Weighted gene co-expression network analysis
