# Supplementary material for: De novo sequencing of the transcriptome reveals regulators of the floral transition in Fargesia macclureana (Poaceae)
Source: BMC Genomics. 2019 Dec 30;20:1035. doi: 10.1186/s12864-019-6418-2 (PMC6937737; doi:10.1186/s12864-019-6418-2)
Supplement: Supplementary file 1 — Additional file 1: Table S1. Statistic of sequencing and assembly data. [file 12864_2019_6418_MOESM1_ESM.docx]

**Table S1**. Statistic of sequencing and assembly data.

| Sample ID | Clean Read | Clean base | GC (%) | N (%) | Q20 (%) | Q30 (%) |
| --- | --- | --- | --- | --- | --- | --- |
| T01 | 21,621,501 | 6,469,541,620 | 53.78 | 0.02 | 95.29 | 89.24 |
| T02 | 20,999,010 | 6,283,275,990 | 54.41 | 0.02 | 95.27 | 89.17 |
| T03 | 22,368,606 | 6,682,897,684 | 54.62 | 0.02 | 95.43 | 89.53 |
| T04 | 24,978,441 | 7,456,250,736 | 55.04 | 0.02 | 95.58 | 89.88 |
| T05 | 23,085,185 | 6,896,780,396 | 55.24 | 0.02 | 95.65 | 89.93 |
| T06 | 21,093,649 | 6,307,722,096 | 54.72 | 0.02 | 95.19 | 89.03 |
| T07 | 24,266,367 | 7,260,090,426 | 54.19 | 0.02 | 95.35 | 89.44 |
| T08 | 22,853,211 | 6,820,270,132 | 54.60 | 0.02 | 95.7 | 90.03 |
| T09 | 23,663,637 | 7,069,596,012 | 54.86 | 0.02 | 95.5 | 89.62 |
| T10 | 24,015,994 | 7,178,431,892 | 54.84 | 0.02 | 95.43 | 89.54 |
| T11 | 23,220,891 | 6,927,588,226 | 55.75 | 0.02 | 95.65 | 89.89 |
| T12 | 23,893,876 | 7,142,273,246 | 54.80 | 0.02 | 95.3 | 89.31 |
| T13 | 23,018,976 | 6,877,524,646 | 54.37 | 0.02 | 95.3 | 89.3 |
| T14 | 23,473,672 | 7,015,134,608 | 54.22 | 0.02 | 95.38 | 89.46 |
| T15 | 22,482,116 | 6,711,336,986 | 55.42 | 0.02 | 95.54 | 89.71 |
| T16 | 42,873,059 | 12,828,515,078 | 55.86 | 0.01 | 96.58 | 91.87 |
| T17 | 41,399,472 | 12,379,427,388 | 55.12 | 0.01 | 96.69 | 92.1 |
| T18 | 42,229,641 | 12,629,576,376 | 54.66 | 0.01 | 96.6 | 91.96 |
| Average | 26,196,517 | 7,829,790,752 | 54.81 | 0.02 | 95.64 | 89.95 |
| Total | 471,537,304 | 1.40936E+11 |  |  |  |  |
