# Supplementary figures and images for: De novo sequencing of the transcriptome reveals regulators of the floral transition in Fargesia macclureana (Poaceae)
Source: BMC Genomics. 2019 Dec 30;20:1035. doi: 10.1186/s12864-019-6418-2 (PMC6937737; doi:10.1186/s12864-019-6418-2)

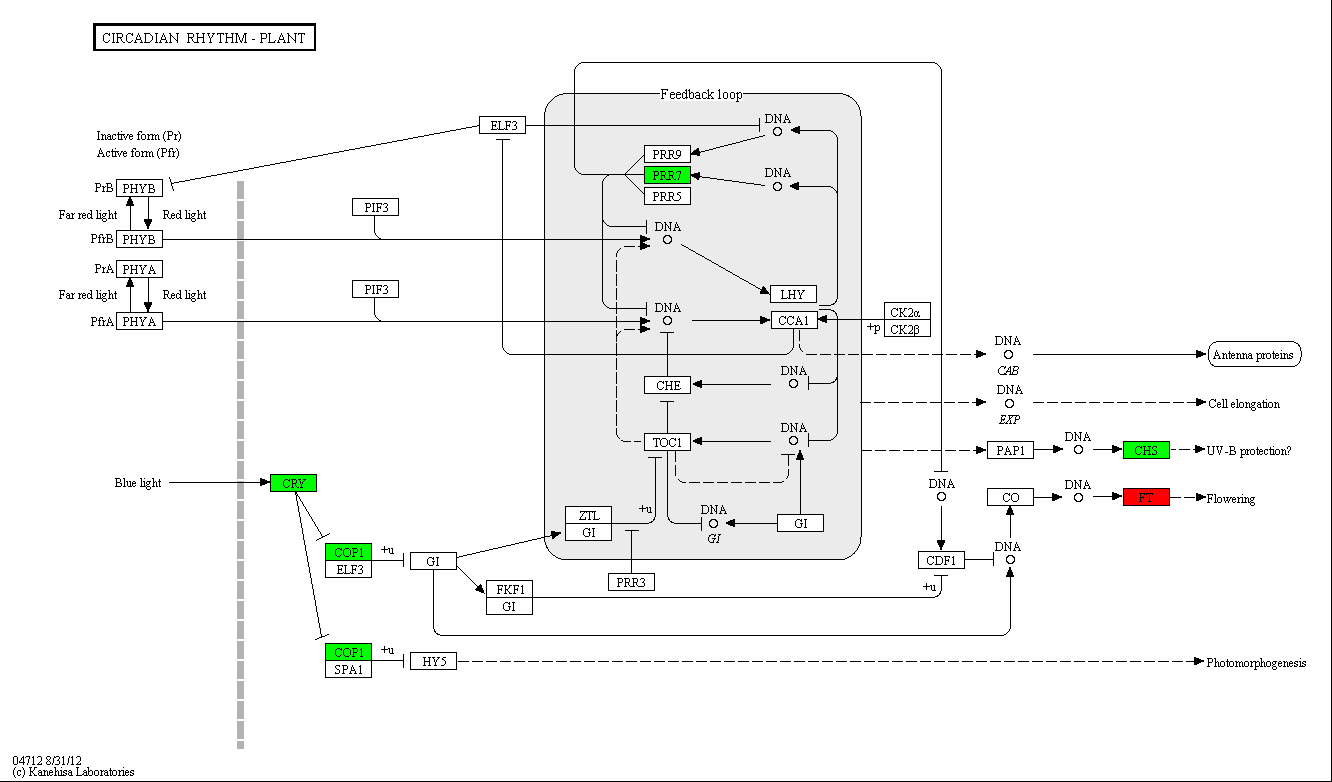

Supplement: Supplementary file 7 — Additional file 7: Figure S2. Hub unigenes in regulatory networks of flowering identified based on analysis of DEUs among tissues. Unigenes c109220.graph_c0 and c110963.graph_c4, showing differential expressions between NF-leaves and F-leaves, are both bamboo orthologs of FLOWERING LOCUS T (FT), which was marked with a red square; while unigenes down-regulated were marked with green squares. [file 12864_2019_6418_MOESM7_ESM.tif]

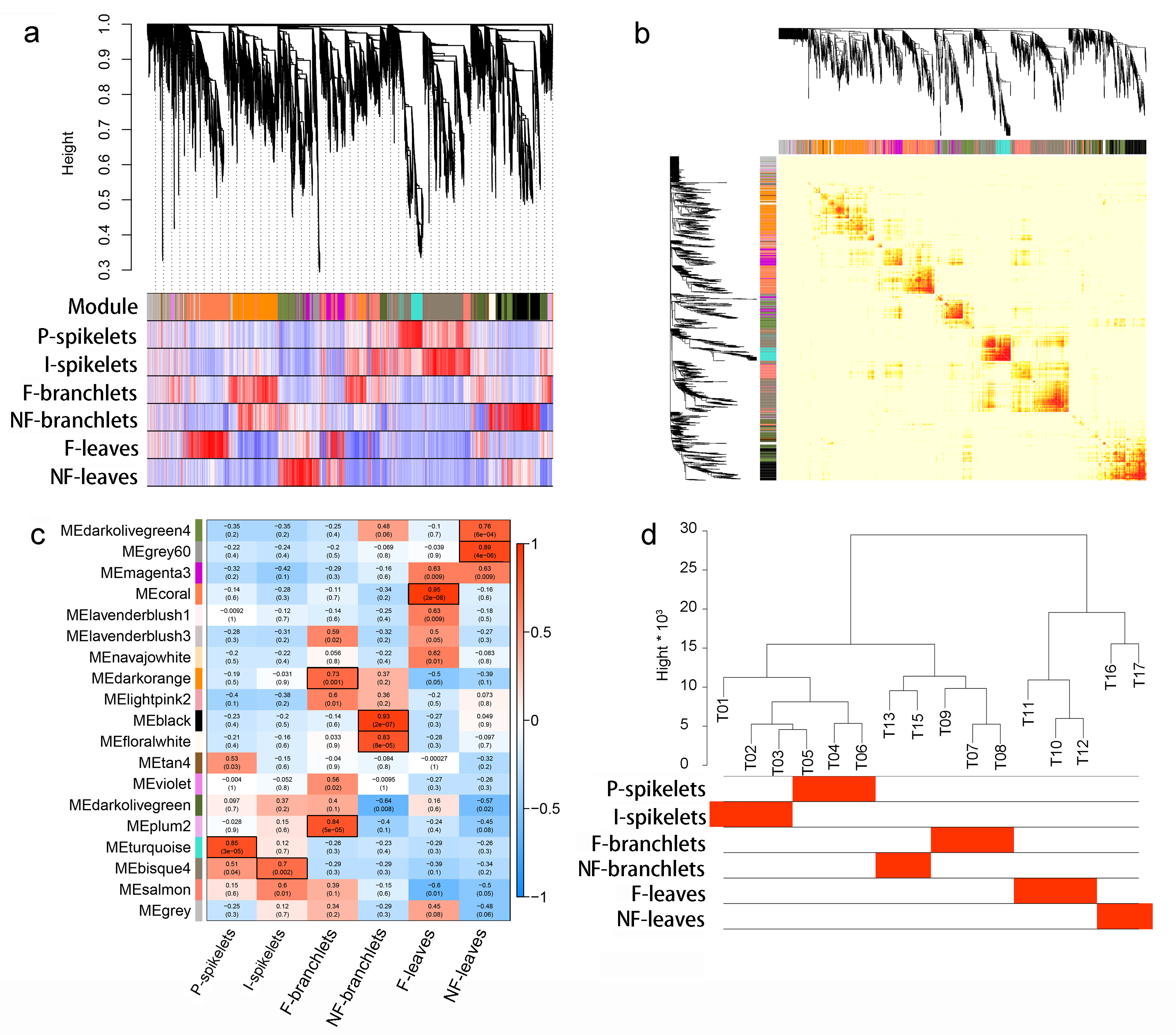

Supplement: Supplementary file 9 — Additional file 9: Figure S3. Weighted gene co-expression network analysis (WGCNA) of all unigenes identified in the transcriptome of F. macclureana. (a) The phylogenetic tree diagram and the heat map related to the traits. This diagram is divided into three parts: the cluster tree of gene system, the module color of corresponding genes, and the correlation between genes related to each trait in tested samples and its module. The redder the color, the more positive the correlation; conversely, blue is negatively correlated. (b) Gene co-expression network heatmaps drawn by randomly selected 1500 genes, in which the left and the upper sides are the symmetrical system clustering tree of gene network/module, and the lower right area indicates the dissimilarity between genes, and the smaller the value is, the darker the color is. (c) Module and trait correlation heat map showing the relationship between a module and a given trait. The closer the correlation between a shape and a module is to the absolute value of 1, it is likely that this trait is related to the module gene work. (d) Systematic clustering tree of samples based on unigenes expressions. [file 12864_2019_6418_MOESM9_ESM.tif]

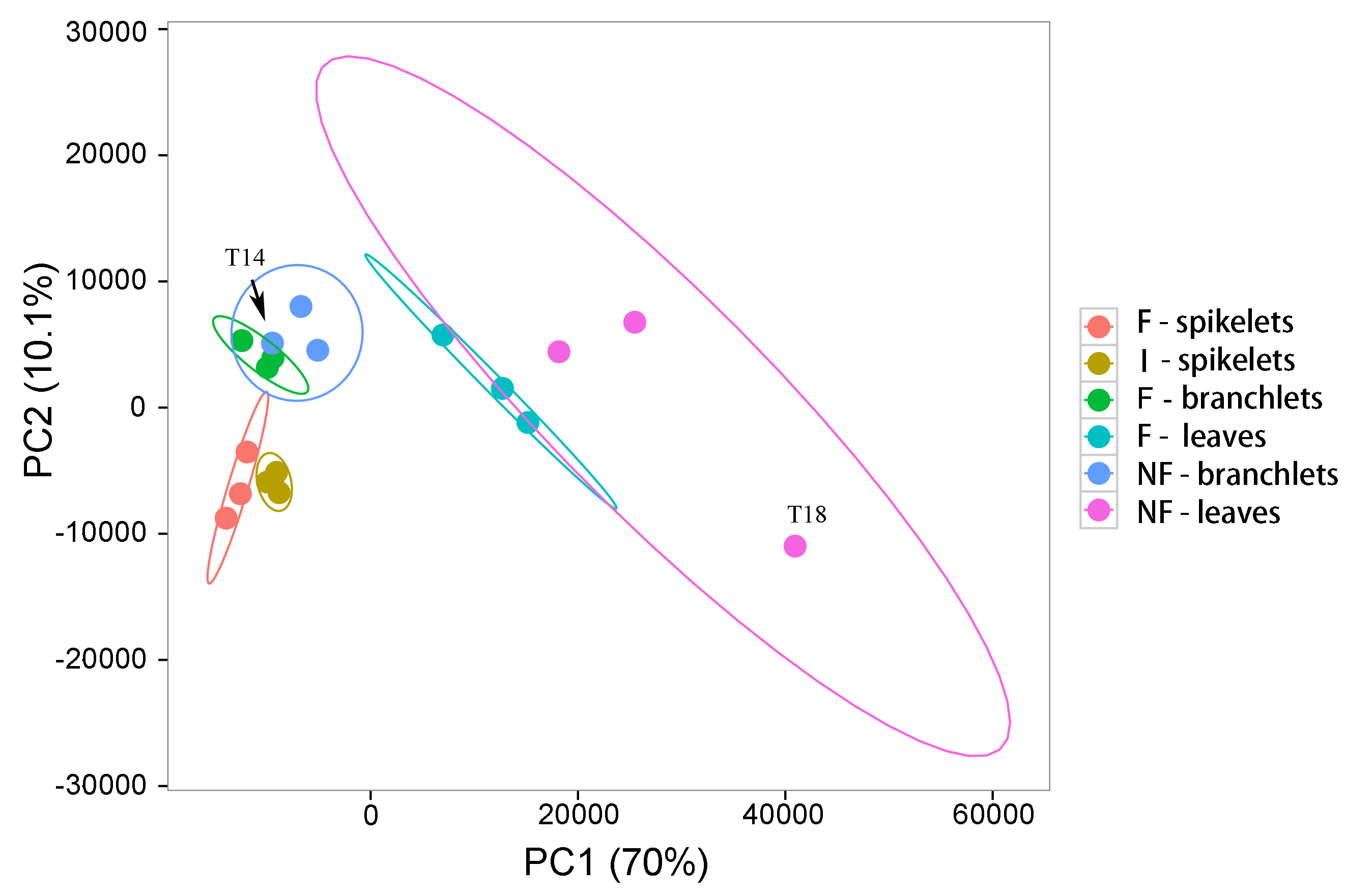

Supplement: Supplementary file 12 — Additional file 12: Figure S4. Principal component analysis (PCA) of unigenes expressions for 18 samples collected from inflorescences in the initial and peak flower stage (I- and P- spikelets), branchlets and leaves of flowering and non-flowering bamboo plants (F/NF-branchlets and F/NF-leaves). [file 12864_2019_6418_MOESM12_ESM.tif]
